# Breast Cancer Patients with Brain Metastases: A Cross-Sectional Study

**DOI:** 10.1155/2022/5763810

**Published:** 2022-08-19

**Authors:** Melih Simsek, Altay Aliyev, Tuba Baydas, Mehmet Besiroglu, Tarik Demir, Abdallah TM Shbair, Mesut Seker, H. Mehmet Turk

**Affiliations:** ^1^BezmialemVakif University, Faculty of Medicine, Department of Medical Oncology, Fatih, Istanbul, Turkey; ^2^BezmialemVakif University, Faculty of Medicine, Department of Internal Medicine, Fatih, Istanbul, Turkey

## Abstract

The prognosis of breast cancer patients with brain metastasis is poor. It was aimed to define the clinicopathological features of breast cancer patients with brain metastases and to determine the risk factors and survival outcomes associated with brain metastasis. This is a single-center, retrospective, cross-sectional study. A total number of 127 patients diagnosed with breast cancer and who developed brain metastasis between January 2011 and March 2021 were retrospectively analyzed. The survival and clinicopathological data of these patients according to 4 biological subtypes were evaluated (luminal A, luminal B, HER-2 overexpressing, and triple-negative). The median overall survival for all patients was 45.6 months. The median time from the diagnosis of breast cancer to the occurrence of brain metastasis was 29.7 months, and the median survival time after brain metastasis was 7.2 months. The time from the diagnosis of breast cancer to brain metastasis development was significantly shorter in HER-2 overexpressing and triple-negative subtypes than in luminal A and B subtypes. The median time from breast cancer diagnosis to brain metastasis was 33.5 months in luminal A, 40.6 months in luminal B, 16.8 months in HER-2 overexpressing, and 22.8 months in the triple-negative groups (*p*=0.003). We found the worst median survival after brain metastasis in the triple-negative group with 3.5 months. Early and close surveillance of high-risk patients may help early diagnosis of brain metastasis and may provide to perform effective treatments leading to longer overall survival times for this patient population.

## 1. Introduction

Breast cancer (BC) remains the most common cancer and the second most common cause of cancer-related death among women worldwide. Although diagnosed in an early stage, the metastatic disease occurs in up to 30% of BC patients [1]. In the course of the disease, brain metastasis (BM) has been seen in 10–15% of the metastatic BC patients [2]. Although the incidence of BM has been 5.2% for early-stage disease in a 10-year follow-up, BM occurs in more than 25% of all BC patients at some point [3]. Due to longer survivals as a result of early detection and efficient treatments such as targeted treatments, the incidence of BM is rising [4]. Advances in imaging modalities also contribute to this increase in incidence [5]. Severe cognitive, sensorial, and neurological impairments and poor prognosis are anticipated in patients with BM [6, 7]. For handling poor outcomes in BM patients, it is a significant challenge to develop management strategies.

After lung cancer, BC is the second most common cause of BM, such that breast and lung cancer metastases are diagnosed more commonly than primary brain tumors [8]. In newly diagnosed BC patients, the median time for BM occurrence is 33 months, and the median survival time after BM is five months [9]. Also, BM may be the first recurrence site in some patients. At this point, the recognition of certain subsets of patients with a tendency for BM will provide us the opportunity for early detection and to take preventive measures in high-risk groups to develop specific targeted therapies that will improve the quality of life and survival outcomes of these patients [10]. The most important risk factors related to the occurrence of BM are younger age, poor performance status, tumor size, grade, nodal status, hormone receptor (HR) status, and disease burden [11]. Chemotherapy responses, survival outcomes, and sites of distant relapses are different among subtypes of BC [12]. These molecular subtypes are based on gene expression profiles such as luminal A, luminal B, HER-2 overexpressing, and triple-negative [2]. It has been shown that these subtypes manifest differently metastasize tendencies for certain locations. While estrogen and progesterone receptor-positive tumors are inclined to metastasize to the bones, HER-2 overexpressing tumors and triple-negative tumors are more likely to metastasize visceral organs including the brain [1].

This study aims to review the clinicopathological features of BC patients with BM, determine the risk factors and survival outcomes associated with BM, and assess the relationship between treatment options and outcomes of BC patients with BM.

## 2. Materials and Methods

In this single-center cross-sectional study, a total number of 127 female BC patients who developed BM between 2011 and 2021 and followed at the department of medical oncology were retrospectively analyzed. This study was organized according to the STROBE checklist. The data were collected from patient files and hospital information system. We carefully examined the demographic data and the pathologic features of the primary tumor, including patient age at diagnosis, tumor type, histologic grade, and ER, PR, and HER-2 status. BM was determined by different imaging modalities such as magnetic resonance imaging and computed tomography. HR and HER-2 status were determined by IHC analysis. Membranous three positivity of HER-2 with IHC and two positivity of HER-2 with IHC confirmed by FISH were accepted as HER-2 positive. Negative or 1+ values of HER-2 with IHC were accepted as HER-2 negative. BC subtypes were determined according to the HR status and HER-2 expression: ER and/or PR positive and HER-2 negative (luminal A); ER and/or PR positive and either HER-2 positive or HER-2 negative with high levels of Ki-67 (luminal B); ER and PR negative and HER-2 positive (HER-2 overexpressing); and triple-negative. Overall survival (OS) was defined as the time between diagnosis of primary BC and the time for the last visit or death. Time to development of BM was defined as the time between primary BC diagnosis and the first diagnosis of BM. Survival time after BM was defined as the time between the development of BM and the time for the last visit or death. All the data obtained were recorded to the data collection form.

Before data collection, approvals from the local ethics committee and institution were obtained for this study. The Helsinki Declaration was complied with in the study. The data were analyzed via Statistical Package for the Social Sciences, version 25 (SPSS v25.0) program (IBM Company). The mean and median values of the variables were calculated by descriptive analysis. Significance was considered as a *p* value of ˂0.05 in a 95% confidence interval. Kruskal–Wallis and Mann–Whitney *U*-tests were performed for in-group and intergroup comparisons.

## 3. Results

A total number of 127 BC patients with BM were included in this study. At the time of BC diagnosis, the median age of the patients was 46.6 (range of 22–73), and the median age of the patients at the time of BM occurrence was 50.9 (range of 29–78). Considering HR status and HER-2 expression, the percentages of subtypes were 6.3% (*n* = 8) for luminal A, 56.7% (*n* = 72) for luminal B, 16.5% (*n* = 21) for HER-2 overexpressing, and 20.5% (*n* = 26) for triple-negative. According to histological subtypes, a large proportion of the patients (92.1%) had invasive ductal carcinoma (*n* = 117), seven patients had invasive lobular carcinoma (5.5%), and only three patients had other subtypes (2.4%).

Of the patients with BM, 33.9% (43/127) had metastatic disease at diagnosis and 6.3% (8/127) had BM at the time of diagnosis. Of the patients with BM, 21.3% (27/127) had also bone metastasis and 60.6% (77/127) had visceral metastasis along with the BM. The median time from primary BC diagnosis to the occurrence of BM was 29.7 months. While 32.3% (41/127) of the patients with BM had solitary metastasis, 7.9% (10/127) had two metastases and 45.7% (58/127) had multiple metastases. Leptomeningeal involvement was observed in 14.2% (18/127) of the patients. After the detection of BM, 90 patients received whole-brain radiotherapy, 19 undergone metastasectomy, and 4 undergone gamma knife surgery. Of the patients, 14 could not receive any treatments for BM because of short survival times. The clinical and histopathological characteristics of the patients and the treatments they had received are summarized in Table 1. The average follow-up time after BC diagnosis to the last contact with the patient either alive or dead was 45.6 months, and the median time from BM occurrence to the last contact with the patient either alive or dead was 7.2 months. After the occurrence of BM, 64.6% (82/127) of the patients died in the first year of BM diagnosis. The 1-year OS rate was calculated as 35.4% for all patients.

In patients with ER and/or PR positivity (80/127), the 1-year OS rate was 37.5%, and the median follow-up period after BM was 7.5 months. Of these patients, 8.8% (7/80) had only BM; 25% (20/80) had also bone metastasis, and 66.2% (53/80) had visceral metastasis among BM. We also examined patients as HER-2-positive and HER-2-negative groups regardless of their hormone status. The median duration of time from BC diagnosis to BM development and the median survival after BM were shorter in the HER-2-positive group than the HER-2-negative group, and the difference was statistically significant (20.4 vs. 37.7 months; *p*=0.017 and 5.3 vs. 11.1 months; *p*=0.014, respectively). Of the HER-2-positive patients, 15.5% (7/45) had only BM, 26.7% (12/45) had additional bone metastasis, and 57.8% (26/45) had visceral metastasis among BM.

**Figure 1:**
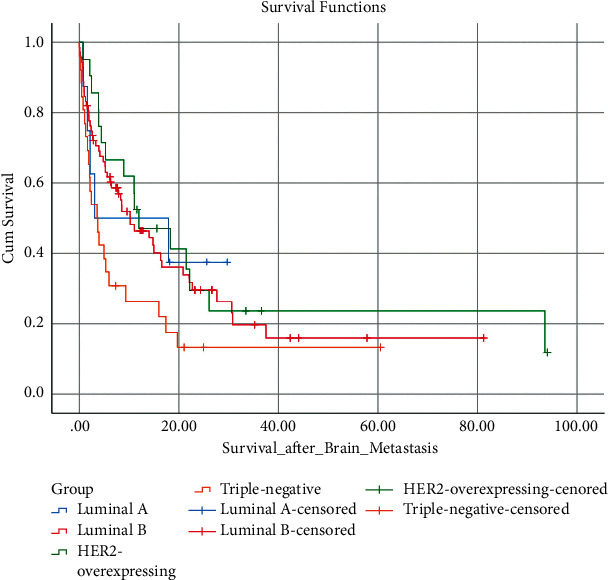
Survival analysis according to breast cancer subtypes.

The median time from BC diagnosis to BM was 33.5 months in the luminal A group, 40.6 months in the luminal B group, 16.8 months in the HER-2 overexpressing group, and 22.8 months in the triple-negative group. A statistically significant difference that originated from HER-2 overexpressing and triple-negative groups was observed between these groups (*p*=0.003). The median survival time after the development of BM was 10.5 months in the luminal A group, 7.6 months for the luminal B group, 11.6 months for the HER-2 overexpressing group, and 3.5 months for the triple-negative group (Figure 1). A statistically significant difference was not observed between these groups (*p*=0.052). The expected survival time after BM was 18.3 months in the group that had only BM, 12 months in the group with additional bone metastasis, and 5.3 months in the group that had BM and visceral metastases. A statistically significant difference originated from visceral metastasis was found between these groups (*p*=0,003). Factors related to the development of BM and the median survival time after BM are summarized in Table 2.

## 4. Discussion

The most common metastasis sites in the clinical course of BC are bone, liver, and brain, occurring 70%, 30%, and 10–30%, respectively [13]. For many BC patients, occurrence of BM has become an important limitation of life expectancy and quality of life 8. The incidence of BM is 14% for HR-positive disease, and the median survival time after BM is 9-10 months. Also, HER-2 positivity, as seen in approximately 25% of BC patients, is related to an increased risk of BM occurrence up to 30–53% for patients with a median survival time of 11–18 months [11, 14]. In this current study, the clinicopathological features of 127 BC patients with BM were evaluated retrospectively.

Both the median time from primary BC diagnosis to the occurrence of BM (29.7 months) and the median time from BM occurrence to the last contact with the patient (7.2 months) were found to be similar to [7, 15]. The average follow-up time after BC diagnosis to the last contact with the patient either alive or dead was 45.6 months.

Tham et al. showed that aggressive pathological features are at a high risk of developing BM [10]. In particular, HER-2-overexpressing and triple-negative subtypes are important risk factors for BM development [16]. Our results were consistent with the literature, for example, the BM development time was significantly shorter in HER-2-overexpressing and triple-negative subtypes compared to luminal subtypes. Ferguson et al. stated that HER-2 overexpression was significantly higher in BM patients [17]. We observed that the HER-2 positive group had a shorter median duration to BM development than the HER-2 negative group (20.4 vs. 37.7 months; *p*=0.018, respectively). Similarly, the median survival after BM diagnosis was statistically shorter in the HER-2 positive group than the HER-2 negative group (5.3 vs. 11.1 months; *p*=0.014, respectively).

As the first site of metastasis, BM was mostly related to the triple-negative subtype and was associated less with HR-positive breast tumors [12]. The triple-negative subtype is known to have shorter survival rates, early relapse, and early BM [18]. The median survival time after BM was shorter for the triple-negative group with 3.5 months in our current study, as reported in several studies ranging between 2.9 and 6.6 months [2, 19]. Similarly, the median survival after BM for our hormone-positive patients was similar to [20]. The coadministration of anti-HER-2 therapies with chemotherapy has improved survival after BM [11]. However, in our study, it was seen that the median survival of the HER-2-positive patients after BM was shorter than in the historical data, which might be due to the limited number of patients. The survival analysis of the breast cancer subtypes is shown in Figure 1.

The recommended treatment for patients with BM is surgical removal when both the metastatic lesion or lesions are resectable and the systemic disease is under control [21, 22]. The survival time after the development of BM was significantly longer in patients with a single metastatic lesion, and this is thought to be associated with curative interventions such as surgical removal or gamma knife therapy. The poor survival of the patients with two or more metastatic lesions might be due to the high number of visceral metastasis, lack of treatments, and the limited number of patients in this subgroup.

## 5. Conclusion

In conclusion, our study demonstrates that HER-2-positive and triple-negative patients have a potentially high risk of BM and a shorter survival trend. Similarly, we observed that patients with multiple metastases or leptomeningeal involvement had poor survivals which might be due to the lack of curative therapeutic interventions. Therefore, early and close surveillance of the high-risk groups might help with the early detection of BM, and this may provide effective treatment for these patients. Also, we must be vigilant in terms of the early recognition of symptoms that occur at the time of BM occurrence.

## Figures and Tables

**Table 1:** Clinical and histopathological characteristics of the patients.

Characteristics		Mean ± SD	Min-max (median)
Age (years)		48.09 ± 11.21	22–73 (46,6)
Overall survival (months)		58.55 ± 51.09	1.8–356 (45.6)
Survival after BM (months)		12.98 ± 16.96	0–94 (7.26)
		*n*	%

Sites of metastasis at BM ^*∗*^development	Only BM^*∗*^	23	18.1
	BM^*∗*^ + bone	27	21.3
	BM^*∗*^ + visceral	77	60.6

Number of BM^*∗*^	Only 1	41	32.3
	2	10	7.9
	>2	58	45.7
	Leptomeningeal	18	14.2

Metastasectomy	Yes	19	15
	No	108	85

Radiotherapy	Whole brain radiotherapy	90	95.7
	Gamma knife	4	4.3

Histology	Invasive ductal	117	92.1
	Invasive lobular	7	5.5
	Others	3	2.4

Receptor status	ER or PR positive	80	63
	ER and PR negative	47	37

HER-2 status	HER-2 positive	45	35.4
	HER-2 negative	82	64.6

Breast cancer subtype	Luminal A	8	6.3
	Luminal B	72	56.7
	HER-2 overexpressing	21	16.5
	Triple negative	26	20.5

Total		127	100

^*∗*^BM: brain metastasis; ER: estrogen receptor; PR: progesterone receptor.

**Table 2:** Factors related to median time between diagnosis of breast cancer and occurrence of brain metastasis and median survival time after brain metastasis.

Variables		Median time for occurrence of BM (months)	Test value *p*	Median survival time after BM (months)	Test value *p*
Sites of metastasis at BM^*∗*^ development	Only BM^*∗*^	20.2		18.3	
	BM^*∗*^ + bone	29.7	3.681^†^	12	11.618^†^
	BM^*∗*^ + visceral	34.9	0.159	5.3	0.003
HER-2 status	Positive	20.4	1371.00^‡^	5.3	1359.00^‡^
	Negative	37.7	0.017	11.1	0.014
Breast cancer subtype	Luminal A	33.5		10.5	
	Luminal B	40.6	13.702^†^	7.6	7.710^†^
	HER-2 overexpressing	16.8	0.003	11.6	0.052
	Triple negative	22.8		3.5	
Receptor positive	Yes	39.8	1169.50^‡^	7.6	1818.50^‡^
	No	19.1	0.000	5.3	0.759

		Median survival time after BM^*∗*^ (months)			Test value *p*
Number of BM^*∗*^	1	12			
	2	2.5			8.296^†^
	Multiple	5.8			0.040
	Leptomeningeal	5.2			
Treatment of BM^*∗*^	Radiotherapy	6.8			
	Surgery	21			36.092^†^
	Gamma knife	20.6			0.000

^*∗*^BM: brain metastasis; ^†^Kruskal–Wallis test; ^‡^Mann–Whitney *U*-test.

## Data Availability

The study of data used to support the findings of this study are available from the corresponding author upon request.
